# Reproductive and Newborn Health

**Published:** 2006-12

**Authors:** Marge Koblinsky

**Affiliations:** Guest Editor, Reproductive and Newborn Health, Public Health Sciences Division, ICDDR,B, GPO Box 128, Dhaka 1000, Bangladesh, Email: margek@icddrb.org, Fax: +880-2-8826050

A new target—universal access to reproductive health by 2015—was endorsed in October 2006 under Millennium Development Goal 5 (MDG-5) to improve maternal health. And while the international reproductive health community could finally celebrate this official recognition of reproductive health on “centre stage of international efforts to defeat poverty and preventable illness” (1), the field reality is far from the target. What does it take to improve sexual and reproductive healthcare practices, including self-care practices at the home and use of services? Generated by a call for papers on these topics, this issue of the Journal contains selected papers describing current practices, examining specific barriers to improved practices, and providing results of interventions aimed at improving self-care practices or use of services. Most practices described relate to improving maternal and newborn* health or care; only two articles provide information on practices in other sexual and reproductive health areas—one on male sexuality and another on women with HIV/AIDS. No papers were received concerning care-seeking for family planning, menstrual regulation, or abortion care—a red flag perhaps signaling the marginalization of these topics in the current day.

Context obviously influences home-care practices. Despite encouraging papers from any region of the world, we still find our major base is here in Bangladesh. Nine of the 17 papers—eight from rural Bangladesh and one from rural Nepal—describe practices in Asia. Six studies cover practices in nine countries of sub-Saharan Africa, and another four studies cover Guatemala, Argentina, Mexico, and Bolivia in Latin America. However, the practices from only one area of Egypt in the Middle East are captured (2). All studies were conducted in rural areas with only two including samples from urban areas (3–4). The immense gap of information about practices in urban areas reflects the wider literature gap of such populations.

Healthcare practices for improved sexual and reproductive health, including maternal and newborn health, are the primary foci of this special issue of the Journal. However, practices and the decision-making behind the practices usually differ depending on whether women, men, or babies are healthy or sick. Papers that describe practices for women and babies most often begin with the practices of healthy persons (5). For example, most newborn-care practices reviewed by Darmstadt *et al.* are the preventive behaviours that should improve the health of the baby (6). Two studies that focus specifically on the illness state include the paper by deBruyn concerned with women with HIV/AIDS and by Killewo *et al.* on practices of rural people with serious illnesses, including maternal complications (7, 8). Contrary to the focus on healthy women and babies, the practices of healthy men of reproductive age are typically not described. Khan *et al.* looked at this group to determine what men's concerns are with regard to their sexual health (3).

For people who are ill and for pregnant women, overcoming barriers to the use of care deemed appropriate by formal medical reviews, should lead to reduction of mortality. However, care-seeking from skilled providers can be low even when women are about to deliver a baby, for various well-known reasons—sociocultural differences, high costs, value women place on delivery by traditional birth attendants (TBA), and chronic under-staffing of health centres (9). Exploring specific barriers, such as costs (10), or barriers for a specific population, such as the poorest (4), enriches our understanding of the barriers and provides insight into means of overcoming them. Parkhurst *et al.* contrast the decision-making for use of facilities for delivery care in Uganda and Bangladesh that helps understand the higher use-rates reported in Uganda (39 vs 12% respectively) (11).

The last section of this issue includes a number of studies on the effects of interventions aimed at improving certain practices and use of services. Community-level interventions to improve practices in the home and use of appropriate services, especially for the ill or for intrapartum care, are typically complex—including training workers for provision of counselling and medications/nutrients, and skills for diagnosis and treatment/referral. At the international level, who that worker is has been contentious but in sites where women are typically outside of services, it could be a traditional birth attendant, a community health worker, or a more skilled community-based or accessible midwife. Through a meta-analysis of 60 studies, Sibley and Sipe explored the effect of TBA training on various practices of women and on perinatal and neonatal mortality, reporting positive results (12). Other studies focused on the package of messages. Whether the package includes messages about birth planning/complication readiness or desired newborn-care practices, is delivered by TBAs or by other community workers, is carried out in Nepal (13), Burkina Faso (14), or rural Bangladesh (15), the results showed significant improvements in many desirable home practices with one major exception: the use of a skilled attendant at birth. Use of skilled birth attendants did not improve in either of the Asian sites, whereas it did in Burkina Faso. This again reflects the variation in use of services for delivery care found more generally between Asian and the African countries. One paper focused on services delivered (active management of third stage of labour) and its cost-efficiency versus expectant management (16).

Only one study focused on referral for needed care of sick newborns, a highly-neglected area (17). This study provides useful insights into the trend of improved behaviours and compliance with referral as the project progresses and trust builds with the advice of community health workers (CHW) and the point of referral. It provides optimism that facilitated referral can work—if the first point of contact i.e. CHW or other community contact persons is considered useful, responsive, and trustworthy.

While all of the above project-level successes are good news, scaling up strategies that focus on home-level practices is a major challenge. Baker's paper on the success of the LINKAGES project to address breastfeeding (both timely initiation and exclusive breastfeeding) at community level in Bolivia (population of one million) and Madagascar (6 million) shows that practices in the home can be changed at scale (18).

Achieving the primary target for MDG 5 by 2015—reduction of maternal mortality by three-quarters—will be next to impossible; achieving universal access to reproductive healthcare should do better. Building the knowledge base for improving access to care is extremely important to achieving such success.

## Footnotes

*Newborn health is not usually included in the definition of sexual and reproductive health. It has been included here as the editor considers integration of the maternal and newborn fields necessary for programming (the mother and baby often use the same care provider)
